# Production of flavonoid *O*-diglycoside naringin via sequential glycosylation in engineered *Schizosaccharomyces pombe* as a whole-cell biocatalyst

**DOI:** 10.5511/plantbiotechnology.26.0125a

**Published:** 2026-06-25

**Authors:** Arisa Kubomura, Takao Ohashi, Ryo Misaki, Kazuhito Fujiyama

**Affiliations:** 1 International Center for Biotechnology, The University of Osaka, 2-1 Yamada-oka, Suita, Osaka 565-0871, Japan

**Keywords:** glucosyltransferase, naringin, rhamnosyltransferase, sequential glycosylation, whole-cell biocatalyst

## Abstract

Some functions of flavonoid glycosides are determined by the glycan structure or enhanced by increasing the attached sugars. Therefore, engineering the glycan moiety can modify the functions of the flavonoid. We attempted to construct a biotransformation method for producing a flavonoid diglycoside, naringin (NRGI) as a model target, using engineered yeast. Naringenin (NRG) glycosylation is initiated by the regiospecific 7-*O*-glucosyltransferase (7-*O*-GlcT) to form naringenin-7-*O*-glucoside (N7G), followed by further rhamnosylation via the branching-type α1,2-rhamnosyltransferase (1,2RhaT), producing NRGI as the final product. In this study, we introduced three α1,2-rhamnosyl-glucoside synthesis-related enzymes—7-*O*-GlcT from *Arabidopsis thaliana* (AtGT-2), 1,2RhaT from *Citrus maxima* (Cm1,2RhaT), and UDP-Rha synthase from *A. thaliana* (AtRHM2)—into the fission yeast *Schizosaccharomyces pombe*. To improve the titer of NRGI, we examined the effects of biotransformation medium composition, initial cell concentration, and cell-permeabilizing reagents. Consequently, we successfully constructed a biotransformation method for producing a flavonoid diglycoside from the aglycone via sequential glycosylation using a single recombinant yeast. Using the optimized biotransformation method, we produced 23.1±1.1 mg l^−1^ of NRGI, 8.0±0.4% molar conversion, from 136 mg l^−1^ of NRG in 24 h cultivation. This study demonstrated the first example of flavonoid *O*-diglycoside production in engineered *S. pombe* via sequential glycosylation by uridine diphosphate sugar-dependent glycosyltransferases (UGTs) under optimized production conditions.

## Introduction

Flavonoids constitute one of the largest specialized metabolite families found in plants and possess a wide range of biological activities. They offer tremendous benefits for human health by virtue of their antioxidative, antibacterial, antiviral, anticancer, and antiallergic activities; they also function as flavor agents (Chouhan et al. 2017; Mrudulakumari Vasudevan and Lee 2020). Their water solubility and high bioavailability in humans (Ji et al. 2020) further make them valuable as nutritional supplements. Structurally, flavonoids share a diphenylpropanes backbone (C6-C3-C6), and are diversified by various modifications, such as hydroxylation, methoxylation, and glycosylation, leading to more than 9,000 known derivatives. Among these, glycosylation is especially prominent in plants, and diverse glycosylation patterns strongly modulate the biological functions of flavonoid glycosides (Kim et al. 2015). For example, naringenin 7-*O*-α1,2-rhamnosyl-glucoside (neohesperidoside), i.e., naringin (NRGI), has a bitter taste (Peterson et al. 2006a), while naringenin 7-*O*-α1,6-rhamnosyl-glucoside (rutinoside), i.e., narirutin, is tasteless (Peterson et al. 2006b). These flavanone glycosides contribute to the characteristic flavors of citrus fruits.

An attractive strategy for producing flavonoid glycosides is the use of engineered microbial hosts expressing uridine diphosphate sugar-dependent glycosyltransferases (UGTs). UGTs, belonging to glycosyltransferase family 1 (GT1) in the carbohydrate-active enzyme (CAZy) database (Lombard et al. 2014), catalyze the biosynthesis of glycoside moieties using diverse UDP-sugars, including UDP-arabinose, UDP-galactose, UDP-glucose (Glc), UDP-glucuronic acid, UDP-rhamnose (Rha), and UDP-xylose, as sugar donors. Several studies have reported microbial biotransformation of flavonoid monoglycosides, especially using *Escherichia coli* and *Saccharomyces cerevisiae* (Isogai et al. 2022). In contrast, only two studies have described diglycosides biotransformation via sequential glycosylation in *E. coli* (An et al. 2016) and *S. cerevisiae* (Xiao et al. 2023).

The ability to design and produce flavonoid glycosides with desired biological functions is highly desirable. This requires regiospecific attachment of sugars to either the aglycone or a glycan. As shown by the example of NRGI and narirutin, differences in the regiospecific attachment of Rha as the second sugar can drastically alter taste. Diglycosylation, compared with monoglycosylation, enables greater structural diversity and increases the likelihood of obtaining flavonoid glycosides with beneficial biological properties. In some cases, multiple glycosylation improves functionality. For example, rutin (quercetin 3-*O*-α1,6-rhamnosyl-glucoside) is more stable in aqueous solution at 100°C (Buchner et al. 2006) and degrades at a slower rate in phosphate buffer containing Fe^2+^ and Cu^2+^ more than quercetin (Makris and Rossiter 2000). From an industrial perspective, completing sequential glycosylation reactions within a single recombinant strain is advantageous compared with co-culturing multiple recombinants, which requires complex optimization (Jones et al. 2016). In contrast, a single engineered strain performing sequential glycosylation reduces the number of variables to controls.

We selected fission yeast *Schizosaccharomyces pombe* as the host for the biotransformation. Yeasts possess favorable bioprocessing traits comparing with *E. coli*, for instance, a larger cell size enabling an easier separation, a lower growth temperature, lower pH and toxic by-product tolerance, no risk of potential phage contamination, and post-translational modification (Liu et al. 2013). *S. pombe* is attractive for recombinant protein production because its cellular processes, such as cell cycle, transcription, translation, and post-translational modifications, are more similar to those of higher eukaryotes, including plants, than those of *E. coli* and *S. cerevisiae* (Takegawa et al. 2009).

**Figure 1:**
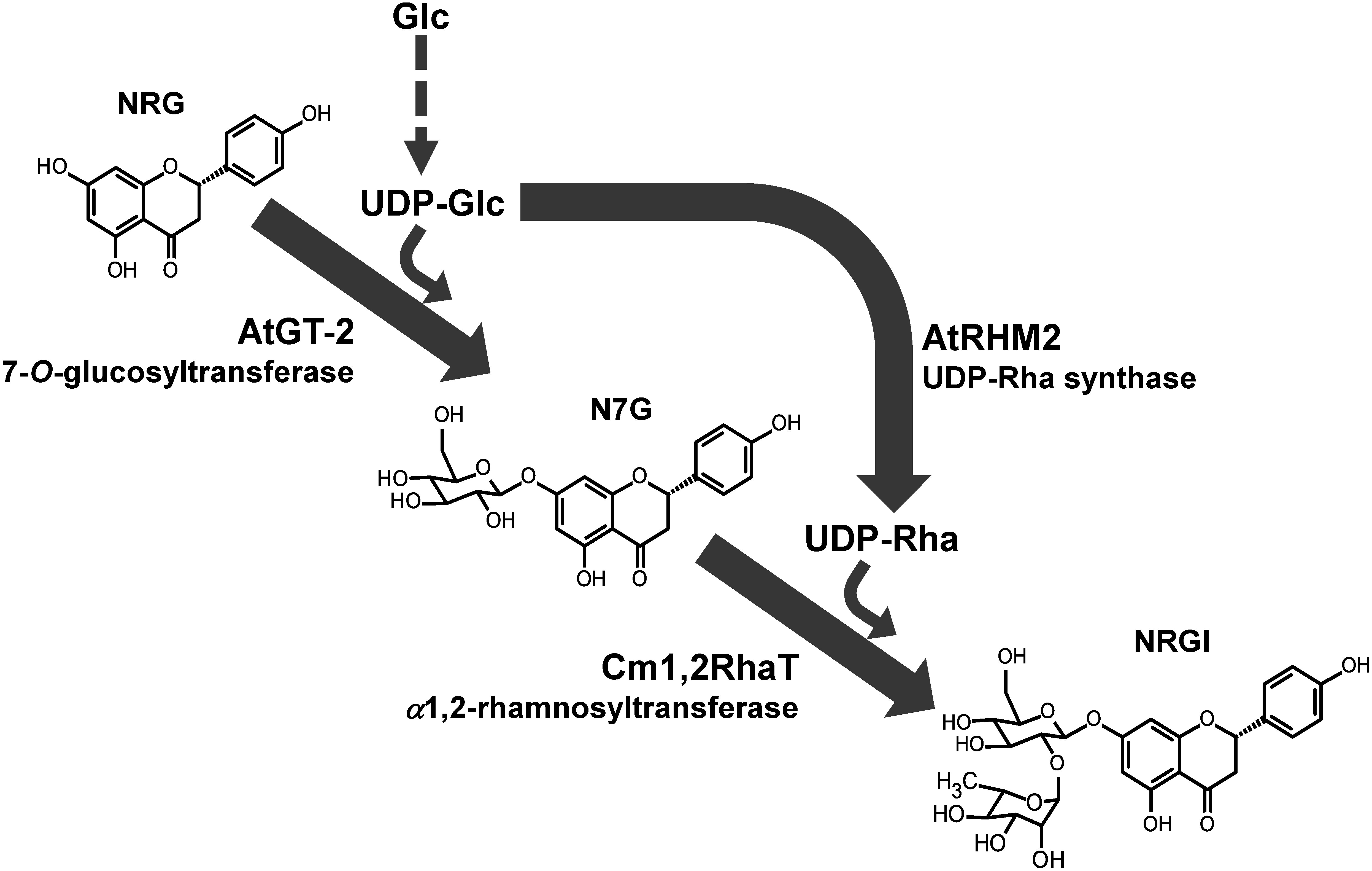
Figure 1. Engineered pathway for NRGI from NRG in *S. pombe*. NRGI is produced from exogenous NRG via sequential glycosylation using AtGT-2, Cm1,2RhaT, and AtRHM2 in engineered *S. pombe* cells. UDP-Glc is synthesized from Glc by the endogenous enzymes.

In this study, we constructed a biosynthesis strategy using engineered *S. pombe* to produce the flavonoid diglycoside NRGI, which has an α1,2-rhamnosyl-glucoside on the aglycone naringenin (NRG), via sequential glycosylation by two UGTs and UDP-Rha synthase (RHM) for the first time (Figure 1). We also examined biotransformation methods to improve productivity in engineered *S. pombe*. In our previous work, we developed an engineered *S. pombe* that could produce a flavonoid diglycoside, NRGI or narirutin, from a flavonoid monoglycoside, naringenin-7-*O*-glucoside (N7G), by introducing citrus rhamnosyltransferases (RhaTs) and RHM (Ohashi et al. 2016). Here, we expanded this platform to use aglycone NRG as a starting substrate. We selected AtGT-2 as a glucosyltransferase because it exhibits broad substrate specificity (Kim et al. 2006). This feature is particularly advantageous for constructing a glycoside biosynthesis system, as AtGT-2 enables the potential production of diverse glycoside compounds from different aglycone substrates. In addition to the UGTs, the introduction of nucleotide sugar biosynthesis machinery was required because UDP-Rha is specific to plants. This system thus enables attachment of Rha moieties and the production of more diverse flavonoid glycans.

## Materials and methods

### Materials

NRG and N7G were purchased from Extrasynthese (Lyon, France). NRGI and 1-deoxynojirimycin were purchased from Wako (Osaka, Japan). UDP-Glc was purchased from Yamasa Corporation (Chiba, Japan). Aureobasidin A (AbA) was purchased from Takara Bio (Shiga, Japan).

### Strains and media

*E. coli* DH5α was used for all cloning procedures. The *S. pombe* strains used in this study were ARC039 (*h*^−^ *leu1-32* *ura4-C190T*) and A8 (*h*^−^ *leu1-32* *ura4-D18 psp3* *isp6* *oma1 ppp16 fma2 sxa2* *atg4 ppp20*::*ura4*^+^) (Idiris et al. 2010) and were grown in standard rich medium (YES, yeast extract/supplement) or synthetic minimal medium (MM) (Moreno et al. 1991). *S. pombe* cells were transformed using the lithium acetate method (Morita and Takegawa 2004).

### Plasmid construction

An *AtGT-2* gene (UGT73B1, At4g34138) (Kim et al. 2006) fragment was prepared from cDNA from *Arabidopsis thaliana*. Total RNA was prepared from the *A. thaliana* flower using the RNeasy Plant Mini kit (QIAGEN, Venlo, Netherlands). The RNA was reverse transcribed into cDNA using a PrimeScript II™ 1st strand cDNA Synthesis Kit (Takara Bio). The open reading frame (ORF) of the *AtGT-2* gene was amplified by PCR using KOD-Plus-NEO DNA polymerase (Toyobo, Osaka, Japan), cDNA from *A. thaliana*, and the following primers: AtGT2-IF-AUR-F, 5′-CCGCTAGGATCTCGAGATGGGAACTCCTGTCGAAGTCTC-3′, and AtGT2-IF-3×FLAG-R 5′-ATGATCTTTGTAATCTACCTTCTCTTTTTGCAGTTTAAC-3′. The PCR products were joined with pGEM®-T Easy Vector (Promega, Madison, WI, USA), and the resulting plasmid was designated pAtGT-2. The ORF of the *AtGT-2* gene was amplified by PCR using KOD-Plus-NEO DNA polymerase (Toyobo), pAtGT-2, and the above-mentioned primers: AtGT2-IF-AUR-F and AtGT2-IF-3×FLAG-R. We prepared a protein expression vector for the *S. pombe*, pAUR224 (Takara Bio), fused with the DNA fragment encoding a 3×FLAG epitope 3′-terminally. The linearized pAUR224-FLAG fragment was amplified by inverse PCR using KOD-Plus-NEO DNA polymerase (Toyobo), pAUR224-FLAG (unpublished data) which harbored the 3×FLAG epitope tag, and the following primers: FLAG-IF-F 5′-GATTACAAAGATCATGATGGTGATTATAAGGATC-3′ and pAUR-IF-R 5′-TCGAGATCCTAGCGGATCTGAC-3′. To construct an *AtGT-2* expression plasmid for *S. pombe*, the amplified *AtGT-2* and the linearized pAUR224-FLAG fragments were joined by the In-Fusion enzyme (Takara Bio), resulting in the pAUR224-AtGT-2-FLAG. To construct an *AtGT-2* expression vector without epitope tag, pAtGT-2 was digested with *Xho*I and *Pst*I and cloned into the equivalent site of pAUR224-FLAG to replace the the 3×FLAG epitope tag with the *AtGT-2* gene, resulting in pAUR224-AtGT-2. The purified plasmids were sequenced by using the BigDye Terminator version 3.1 cycle sequencing kit and a 3130xl genetic analyzer (Applied Biosystems, Waltham, MA, USA). Previously constructed plasmids pREP2-AtRHM2-MycHis and pREP1-Cm1,2RhaT-MycHis were used for the biotransformation of NRGI (Ohashi et al. 2016).

### SDS-PAGE analysis

*S. pombe* transformants were cultured in YES or MM without leucine and uracil (MM-leu-ura) containing 0.5 µg ml^−1^ of AbA at 30°C for 48 h. The cultured cells were harvested, resuspended in 200 µl of alkaline extraction buffer containing 0.1 M NaOH, 50 mM EDTA, 2% sodium dodecyl sulfate (SDS), and 2-mercaptoethanol, heated at 95°C for 5 min, and neutralized with 5 µl of 4 M acetic acid. After the addition of bromophenol blue, the cell lysate sample was separated by 10% SDS-PAGE and electroblotted onto a polyvinylidene difluoride membrane (Merck Millipore, Darmstadt, Germany). Immunodetection analysis was conducted using mouse monoclonal Anti-FLAG M2 antibody (Sigma-Aldrich, St. Louis, MO, USA) with a dilution of 1 : 10000 for AtGT-2 protein or mouse anti-c-Myc antibody (Invitrogen, Waltham, MA, USA) with a dilution of 1 : 10000 for AtRHM2 and Cm1,2RhaT proteins as primary antibodies and horseradish peroxidase-conjugated sheep anti-mouse whole antibody (GE Healthcare, Chicago, IL, USA) as the secondary antibody with a dilution of 1 : 10000. FLAG- and c-Myc-specific signals were visualized by enhanced chemiluminescence (Immobilon Western; Merck Millipore).

### In vitro glycosylation assay using crude soluble protein

Preparation of crude soluble proteins and glycosylation assays were conducted as described previously with slight modifications (Ohashi et al. 2016). Briefly, for N7G production, *S. pombe* ARC039 transformants harboring pAUR224-AtGT-2-FLAG or pAUR224-AtGT-2 were grown in 100 ml of YES containing 0.5 µg ml^−1^ of AbA at 30°C for 24 h. For NRGI production, *S. pombe* A8 transformants harboring pAUR224-AtGT-2, pREP1-Cm1,2RhaT-MycHis, and pREP2-AtRHM2-MycHis were grown in 100 ml of MM-leu-ura containing 0.5 µg ml^−1^ of AbA at 30°C for 24 h. The cultured cells were harvested and resuspended in 1 ml of 50 mM phosphate buffer (pH 7.5) supplemented with the cOmplete EDTA-free protease inhibitor cocktail (Roche Applied Science, Mannheim, Germany), and cells were homogenized with glass beads. The mixture was vortexed for 15 s followed by 30 s on ice and this cycle was repeated 20 times. The mixture was centrifuged at 10,000×g for 10 min and the resultant supernatant was used for enzyme assays. For N7G production, standard enzyme assays were performed in 50 mM phosphate buffer (pH 7.5) containing the cOmplete EDTA-free protease inhibitor cocktail (Roche). The reaction mixture contained 150 µM flavonoid substrate, 500 µM UDP-Glc, 250 µM 1-deoxynojirimycin, and 4 µl of extracted crude protein in a total volume of 20 µl. After incubation for 30 min at 30°C, the reaction was terminated by the addition of 20 µl of methanol. Screening experiments of optimal pH and temperature were conducted in the standard enzyme assays and incubated for 1 h. For NRGI production, standard enzyme assays were performed in 50 mM phosphate buffer (pH 7.5) containing the cOmplete EDTA-free protease inhibitor cocktail (Roche). The reaction mixture contained 150 µM flavonoid substrate, 500 µM UDP-Glc, 250 µM 1-deoxynojirimycin, 3 mM NAD^+^, 3 mM NADPH, and 4 µl of extracted crude protein in a total volume of 20 µl. After incubation for 16 h at 30°C, the reaction was terminated by the addition of 20 µl of methanol. The reaction products were analyzed by reversed-phase high-performance liquid chromatography (RP-HPLC). The amounts of the products were calculated from the peak areas and taken an average from three independent experiments.

### Biotransformation of NRG using engineered *S. pombe*

For N7G production, *S. pombe* ARC039 transformants harboring pAUR224-AtGT-2 were pre-cultured in 100 ml of YES containing 0.5 µg ml^−1^ AbA at 120 rpm at 30°C for 24 h. Cells were harvested by centrifugation at 5,000×g for 10 min and washed with distilled water. Cells were cultured either in YES medium or in phosphate buffer supplemented with 40 g l^−1^ glucose, both containing 500 µM NRG and 250 µM 1-deoxynojirimycin, and adjusted to OD_600_ of 1 or 33, respectively. Both biotransformation media were agitated at 220 rpm at 30°C for 48 h. For the repetitive N7G production, the first batch was cultured for 24 h under the same conditions as the phosphate buffer described above. After the first batch, cells and supernatant were separated by centrifugation. The second batch was initiated by resuspending the harvested cells in fresh phosphate buffer, supplemented with 40 g l^−1^ glucose, 500 µM NRG, and 250 µM 1-deoxynojirimycin. This was continued through the third batch. For NRGI production, *S. pombe* A8 transformants harboring pAUR224-AtGT-2, pREP2-AtRHM2-MycHis, and pREP1-Cm1,2RhaT-MycHis were precultured in 100 ml of MM-leu-ura containing 0.5 µg ml^−1^ of AbA. The precultured cells were cultured for 48 h under the same conditions as the phosphate buffer described above. The supernatant was collected and an equal volume of methanol was added to terminate the production. The supernatant sample was further centrifuged at 13,000×g for 10 min to remove culture-derived debris and was then analyzed by RP-HPLC. The amounts of the products were calculated from the peak areas and taken an average from three independent experiments.

### HPLC analysis

RP-HPLC analysis was conducted using Shimadzu Prominence HPLC system (Shimadzu, Kyoto, Japan) and a COSMOSIL 5C18-MS column (4.6 mm×150 mm, Nacalai Tesque, Kyoto, Japan). The mobile phase consisted of solvent A (0.1% v/v formic acid in Milli-Q water) and solvent B (acetonitrile). The gradient elution was programmed as follows: 10% B at 0 min, 40% B at 10 min, 50% B at 20 min, 10% B at 25 min, and maintained at 10% B until 30 min. The flow rate was set at 1.0 ml min^−1^, and the detection was performed at a wavelength of 290 nm.

### Statistical analysis

An unpaired Welch’s *t*-test was used for comparisons between two independent groups. For the in vitro determination of the optimal pH and temperature, statistical analysis was performed using one-way analysis of variance (ANOVA) followed by Tukey’s multiple comparison test. For experiments examining the effects of cell-permeabilizing reagents on N7G production, Dunnett’s test was applied.

## Results

### Enzymatic characterization of AtGT-2

**Figure 2:**
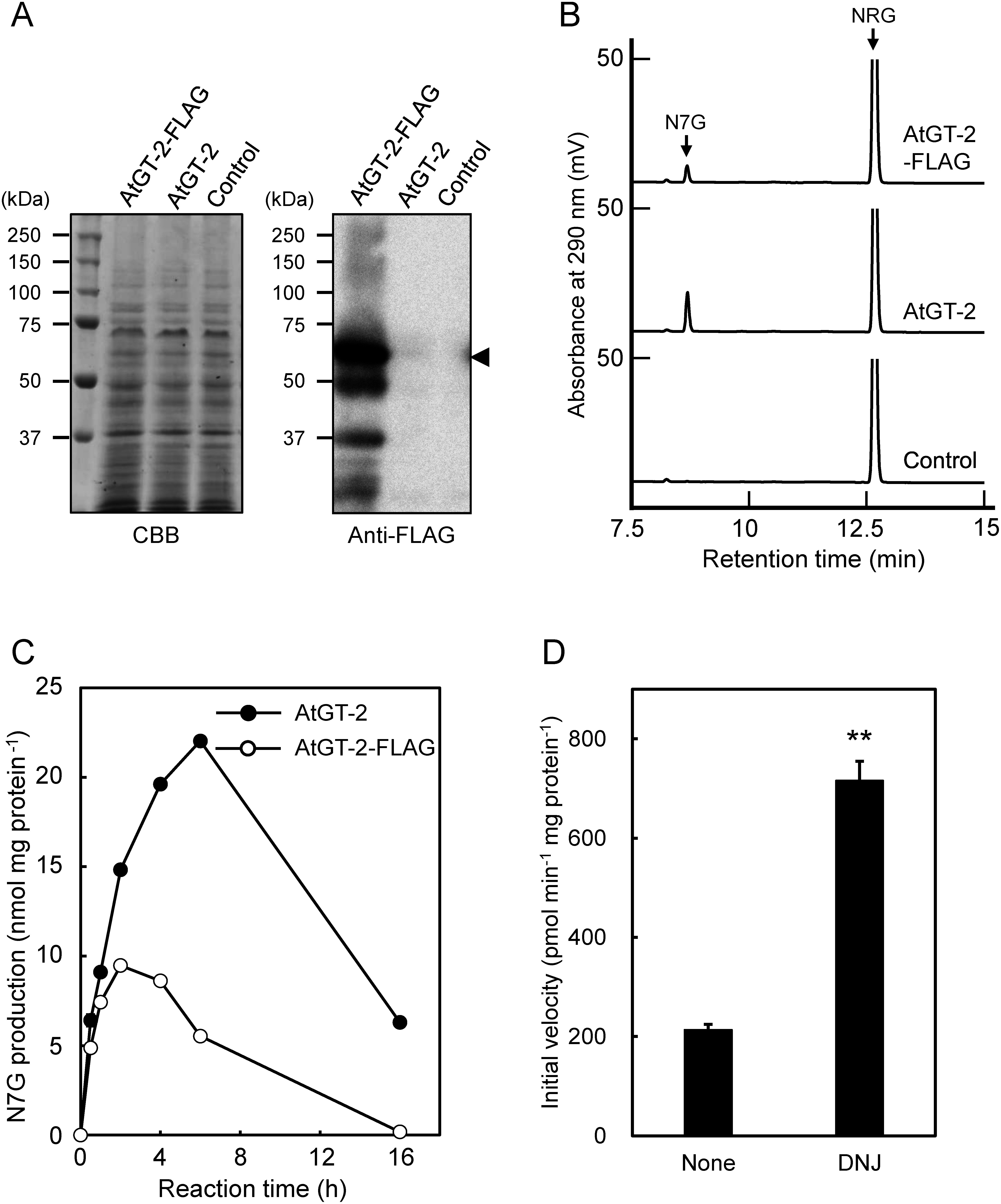
Figure 2. In vitro production of N7G from NRG using soluble protein from engineered *S. pombe* expressing AtGT-2. (A) Immunoblot analysis of recombinant AtGT-2 protein. Crude lysate from *S. pombe* cells harboring pAUR224-AtGT-2-FLAG, pAUR224-AtGT-2, or pAUR224 (control) was subjected to 10% SDS-PAGE and detected by either Coomassie brilliant blue (CBB) staining or immunoblotting using anti-FLAG antibody. Triangle shows specific band corresponding to AtGT-2-FLAG. (B) In vitro enzymatic assay of the AtGT-2 protein. The HPLC profiles of the reaction mixtures using NRG as the acceptor substrate and the prepared crude soluble protein from *S. pombe* cells harboring pAUR224-AtGT-2-FLAG, pAUR224-AtGT-2, or pAUR224 (control) are shown. Arrows on the chromatogram indicate the elution positions of N7G and NRG. All chromatograms are on the same y-axis scale. (C) Time course for in vitro N7G production by AtGT-2 and AtGT-2-FLAG. Amounts of N7G produced in the reaction mixtures at each time point were measured by HPLC. (D) Effects of glucosidase inhibitor on in vitro N7G production using the prepared crude soluble protein from *S. pombe* cells harboring pAUR224-AtGT-2. 1-Deoxynojirimycin (DNJ) was used as a glucosidase inhibitor. Statistical analysis was performed using an unpaird Welch’s *t*-test, with significant differences indicated by ** *p*<0.01.

**Figure 3:**
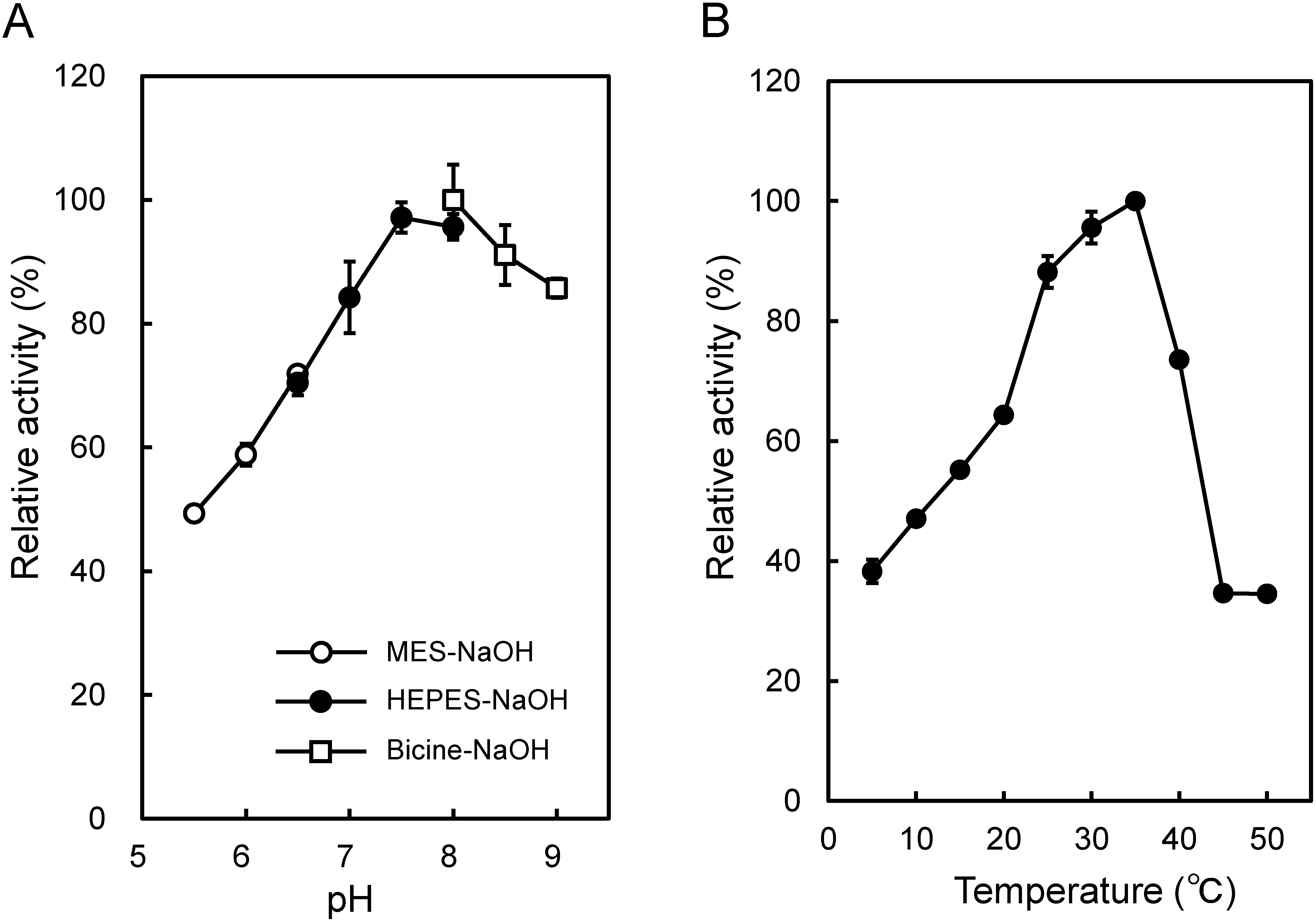
Figure 3. Enzymatic characterization of recombinant AtGT-2. (A) Effect of pH on glucosylation activity toward N7G. The concentrations of MES for pH 5.5–6.5, HEPES for 6.5–8.0, and Bicine for 8.0–9.0 used in the reaction were 100 mM. Glucosylation activity at pH 8.0 was taken as 100%. (B) Effect of temperature on glucosylation activity toward N7G. The reaction mixture was incubated at 5–50°C. Glucosylation activity at 35°C was taken as 100%.

To produce NRGI from NRG, N7G needs to be produced by a glucosyltransferase prior to the rhamnosylation reaction by an α1,2-rhamnosyltransferase (1,2RhaT), which uses N7G as a sugar acceptor. We selected AtGT-2 (At4g34138) as a 7-*O*-glucosyltransferase (Kim et al. 2006) and constructed an *S. pombe* expression vector, namely pAUR224-AtGT-2-FLAG, expressing the recombinant AtGT-2 enzyme as a C-terminally 3×FLAG tagged fusion protein for immunoblot detection. An *S. pombe* expression vector without an epitope tag was also constructed, namely pAUR224-AtGT-2. Total soluble proteins from *S. pombe* ARC039 transformants harboring pAUR224-AtGT-2-FLAG or pAUR224-AtGT-2 were extracted and analyzed by SDS-PAGE and immunoblotting (Figure 2A). In the immunoblot analysis, the specific major bands of a 56.5-kDa protein corresponding to AtGT-2-FLAG were detected only in the transformants harboring pAUR224-AtGT-2-FLAG. The bands of low molecular weight were presumed to be degradation products. Next, we examined glucosylation activity using the prepared crude soluble protein from each transformant, NRG, and UDP-Glc as substrates. HPLC analysis of the enzymatic products showed the newly appearing peak co-eluting with the standard N7G (Figure 2B), indicating the successful production of enzymatically active AtGT-2 in *S. pombe*. The time course of AtGT-2 enzymatic reactions is shown in Figure 2C. The amounts of N7G increased linearly with the reaction time until 1 h for both crude enzymes. The apparent initial velocities of the AtGT-2-FLAG and the AtGT-2 with UDP-Glc toward N7G were 162.6±1.9 and 213±10.8 pmol min^−1^ mg protein^−1^, respectively, indicating that C-terminally-fused FLAG tag caused a 0.76-fold reduction in glucosylation activity. Because crude extracts were used, differences in activity may not be solely attributable to tagging. Nevertheless, the C-terminal tag likely contributed to reduced enzymatic activity. The C-terminals of UGTs commonly share a highly conserved motif, known as the plant secondary product glycosyltransferase (PSPG) motif, which accommodates UDP-sugars. C-terminal tagging might prevent the UDP-sugars from reaching the enzymes. Homology modeling and molecular dynamics simulations would help elucidate this mechanism. Accordingly, AtGT-2 crude extract was used in subsequent experiments. In addition, after 16 h of reaction, the amounts of produced N7G decreased. Because endogenous β-glucosidases in *S. pombe* might be responsible, we tested the addition of 1-deoxynojirimycin as a β-glucosidase inhibitor in the reaction mixture. The apparent initial velocity of the AtGT-2 increased to 716.3±38.4 pmol min^−1^ mg protein^−1^ with 1-deoxynojirimycin (Figure 2D). Glucosidase activity was observed in the culture supernatant of *S. cerevisiae* (Schmidt et al. 2011; Wang et al. 2016; Werner and Morgan 2009). Based on this observation, we hypothesized a similar phenomenon in *S. pombe* and therefore included 1-deoxynojirimycin in biotransformation experiments to improve production. The optimal pH and temperature for AtGT-2 were approximately pH 7.5–8.5 and around 35°C, respectively, based on statistical analysis using one-way ANOVA followed by Tukey’s multiple comparison test (*p*<0.05) (Figure 3A, B).

### Biotransformation of NRG using AtGT-2-expressing *S. pombe* strain to produce N7G

**Figure 4:**
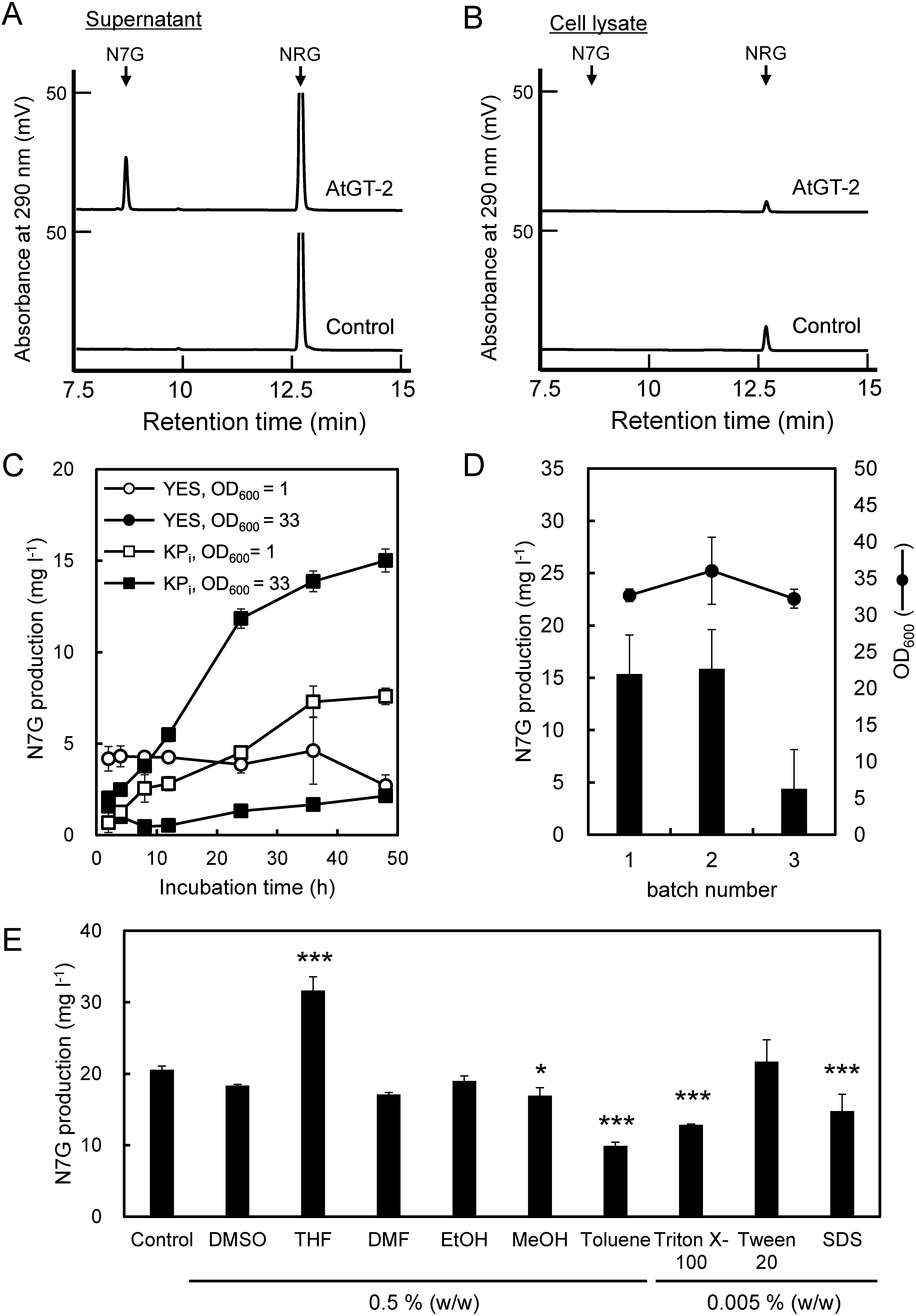
Figure 4. Biotransformation of NRG for the production of N7G in engineered *S. pombe*. (A, B) HPLC analysis of the biotransformation products from culture supernatant (A) and cell lysate (B) of *S. pombe* cells harboring pAUR224-AtGT-2 or pAUR224 (control). Cell lysate was prepared by the glass bead method. Arrows on the chromatograms indicate the elution positions of N7G and NRG, respectively. All chromatograms are on the same y-axis scale. (C) Effects of the biotransfomation medium and the initial cell concentration on the biotransformation of NRG toward the production of N7G in *S. pombe* cells harboring pAUR224-AtGT-2. KP_i_ represents 100 mM potassium phosphate buffer (pH 7.5). (D) N7G production by the repeated batch culture in the engineered *S. pombe*. OD_600_ values were measured and the culture supernatant was collected in each batch. The amount of N7G produced in each supernatant was measured by HPLC. The next round of batch biotransformation was initiated by suspension with phosphate buffer containing Glc, NRG, and 1-deoxynojirimycin using harvested *S. pombe* cells. (E) Effects of cell-permeabilizing reagents on N7G production using *S. pombe* cells harboring pAUR224-AtGT-2. Amounts of N7G produced in the supernatant after 24 h reactions were measured by HPLC. Control represents biotransformation medium containing neither organic solvents nor surfactants. Statistical analysis was performed using Dunnett’s test, with significant differences indicated by * *p*<0.05, ** *p*<0.01, *** *p*<0.001.

We next produced N7G from NRG using the AtGT-2-expressing *S. pombe* strain as a whole-cell biocatalyst. To biotransform NRG, pre-cultured AtGT-2-expressing *S. pombe* cells were inoculated at OD_600_=1 into YES medium supplemented with 500 µM NRG and 250 µM 1-deoxynojirimycin and agitated for 48 h. To examine whether the biotransformation products had accumulated in the cells or been secreted into the medium, both cell lysates and culture supernatant samples were prepared. HPLC analysis of the biotransformation products demonstrated that N7G was produced only in the culture supernatant from AtGT-2-expressing *S. pombe* transformants (2.7±0.6 mg l^−1^, 0.9±0.2% of the molar conversion) but not from the control transformants (Figure 4A). In addition, N7G accumulated in neither the *AtGT*-2-expressing *S. pombe* transformant cells nor the control transformant (Figure 4B).

### Culture process to improve the N7G titer

To improve the N7G titer, we examined the effects of initial cell concentration and medium composition on the biotransformation of NRG into N7G (Figure 4C). YES or phosphate buffer was used for the biotransformation medium. The initial yeast concentration in phosphate buffer was chosen based on a previous study (Raimondi et al. 2011) and our preliminary experiment (Supplementary Figure S1). When the *S. pombe* transformants were cultured in phosphate buffer with an initial cell concentration of OD_600_=33, the highest titer of N7G (15.0±0.6 mg l^−1^, 5.2±0.2% molar conversion) was achieved at 48 h. Prior to this experiment, the initial cell concentration in phosphate buffer was examined at OD_600_=17, 33, and 66 (Supplementary Figure S1). In phosphate buffer, cell density remained stable throughout biotransformation (Supplementary Figure S1B). However, cultures inoculated at OD_600_=66 showed a decline in viability during cultivation. Among these three conditions, OD_600_=33 showed the maximum production at 36 h incubation (27.0 mg l^−1^, 12.5% molar conversion). To further explore the potential of the biotransformation, repetitive batch biotransformation reactions were conducted by recycling the *S. pombe* transformant cells after harvesting the culture supernatant (Figure 4D). In suspension cultures, N7G titers in the culture supernatant were almost comparable until the second batch biotransformation reactions, as were the OD_600_ values, while both were decreased in the third batch. The engineered *S. pombe* exhibited operational robustness, maintaining N7G production over repeated batch culture.

### Enhancement of cell permeability in biotransformation

In a previous report, improving cell permeability in *S. cerevisiae* led to an increased product concentration in whole-cell catalysis (Li et al. 2016). To enhance secretion of intracellularly produced N7G by the increasing cell permeability, we supplemented cell-permeabilizing reagents and examined their effects on the titer of N7G in the supernatant (Figure 4E); dimethyl sulfoxide (DMSO) is used to enhance the cellular permeability (Adams 1972; Georgopapadakou and Smith 1985); tetrahydrofuran (THF) and *N*,*N*-dimethylformamide (DMF) are potential cell membrane-disturbing organic solvents; ethanol and methanol enhance membrane fluidity (Ishmayana et al. 2017); toluene and Triton X-100 enhance the secretion of biotransformation products in an engineered *S. cerevisiae* strain (Li et al. 2016); and Tween 20, SDS are surfactants for the cell lysis and the solubilization of hydrophobic protein. Organic solvents or surfactants were added to the biotransformation medium to reach the final concentrations of 0.5% (w/w) or 0.005% (w/w). Because surfactants at 0.5% (w/w) caused foam while shaking incubation, they were applied at 0.005% (w/w). THF and Tween 20 enhanced the secretions of N7G into the culture supernatant by 1.5 and 1.1-fold respectively compared with the control. Biotransformation with THF for 24 h resulted in the highest N7G titer (31.6±2.0 mg l^−1^, 14.6±0.9% molar conversion).

### In vitro NRGI production via sequential glycosylation using crude soluble protein preparation from the engineered *S. pombe* strain

**Figure 5:**
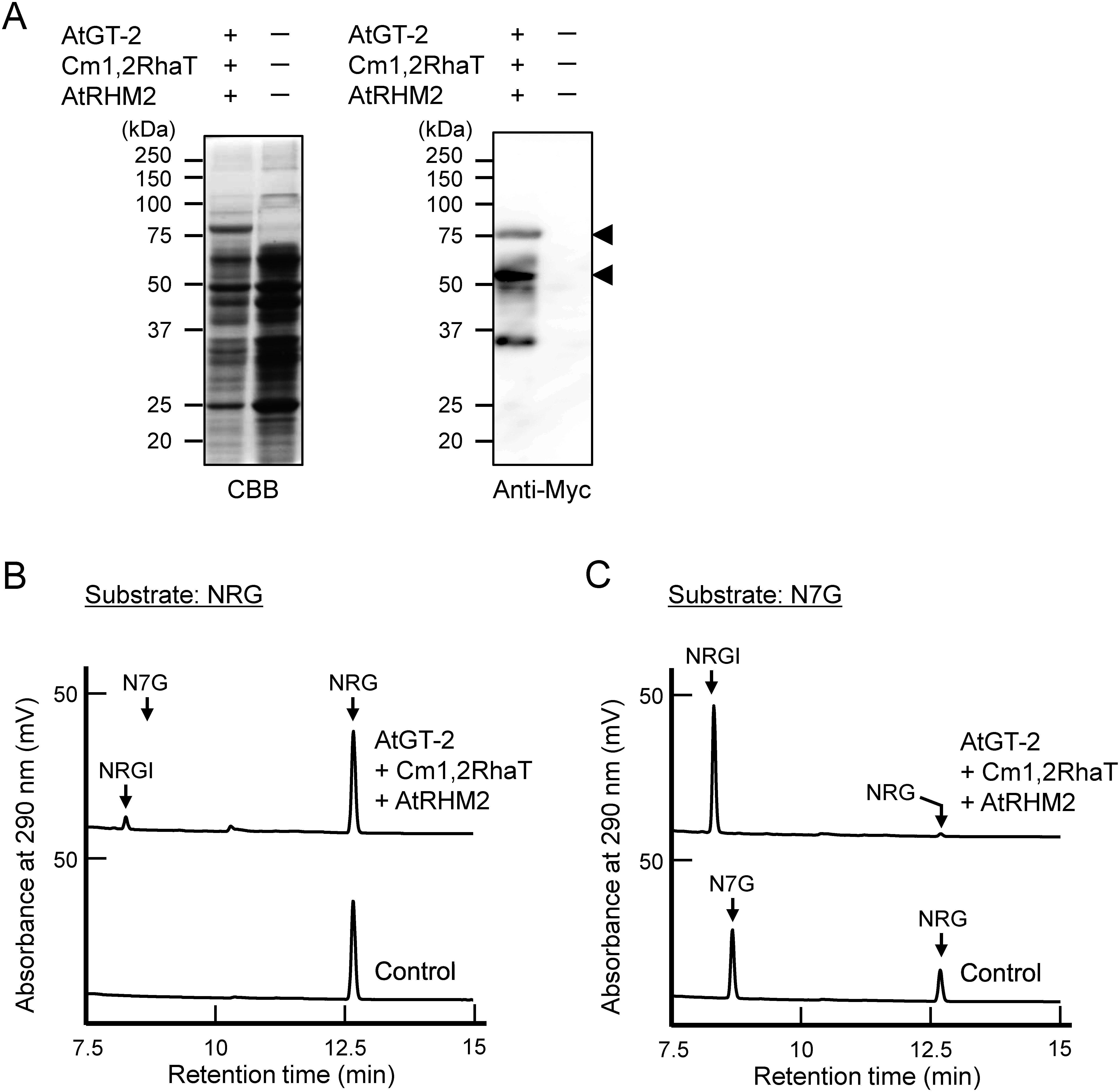
Figure 5. In vitro production of NRGI from NRG using soluble protein from engineered *S. pombe* co-expressing AtGT-2, Cm1,2RhaT, and AtRHM2. (A) Immunoblot analysis of recombinant Cm1,2RhaT and AtRHM2 proteins. Crude lysate from *S. pombe* cells harboring either pAUR224-AtGT-2, pREP1-Cm1,2RhaT-MycHis, and pREP2-AtRHM2-MycHis or pAUR224, pREP1-MycHis, and pREP2-MycHis (control) was subjected to 10% SDS-PAGE and detected by either Coomassie brilliant blue (CBB) staining or immunoblotting using anti-Myc antibody. Triangles show specific bands corresponding to Cm1,2RhaT and AtRHM2. (B, C) In vitro production of NRGI using NRG (B) or N7G (C) and crude protein of *S. pombe* cells harboring either pAUR224-AtGT-2, pREP1-Cm1,2RhaT-MycHis, and pREP2-AtRHM2-MycHis or pAUR224, pREP1-MycHis, and pREP2-MycHis (control). Arrows on the chromatograms indicate the elution positions of NRGI, N7G, and NRG, respectively. All chromatograms are on the same y-axis scale.

The efficient co-expressions of GlcT, RhaT, and UDP-Rha synthase genes are needed to produce NRGI from NRG by biotransformation via sequential glycosylation using the engineered *S. pombe*. Therefore, we used a multiprotease-deficient *S. pombe* A8 strain to reduce the potential protein degradation (Idiris et al. 2010). An *S. pombe* A8 transformant harboring pAUR224-AtGT-2, pREP1-Cm1,2RhaT-MycHis, and pREP2-AtRHM2-MycHis was constructed, and their recombinant protein expressions were analyzed by immunoblot analysis. The specific major bands of the 53.2- and 76.2-kDa proteins corresponding to Cm1,2RhaT and AtRHM2 were detected, respectively, while the band corresponding to AtGT-2 protein was not detected due to the absence of Myc tag. A band around 37 kDa is probably a degradation product (Figure 5A). To examine enzymatic activity, in vitro glucosylation and rhamnosylation assays were conducted using the prepared crude soluble protein, 150 µM of NRG or N7G, and 500 µM of UDP-Glc. HPLC analysis of the enzymatic products from NRG and N7G showed newly appearing peaks co-eluting with the standard NRGI, indicating that those recombinant proteins were enzymatically active and successfully produced NRGI (Figure 5B, C). The intermediate N7G was not observed in the culture supernatant when treated with NRG (Figure 5B). A minor peak at a retention time of 10.3 was observed, which is presumed to be a by-product. Since its retention time did not correspond to any of the reference standards, its identity could not be determined. The titers of NRGI from NRG and N7G after 16 h reactions were 26.2±0.1 and 209.1±8.8 nmol mg protein^−1^, respectively.

### Biotransformation of NRG via sequential glycosylation using a UGT-co-expressing *S. pombe* strain to produce NRGI

**Figure 6:**
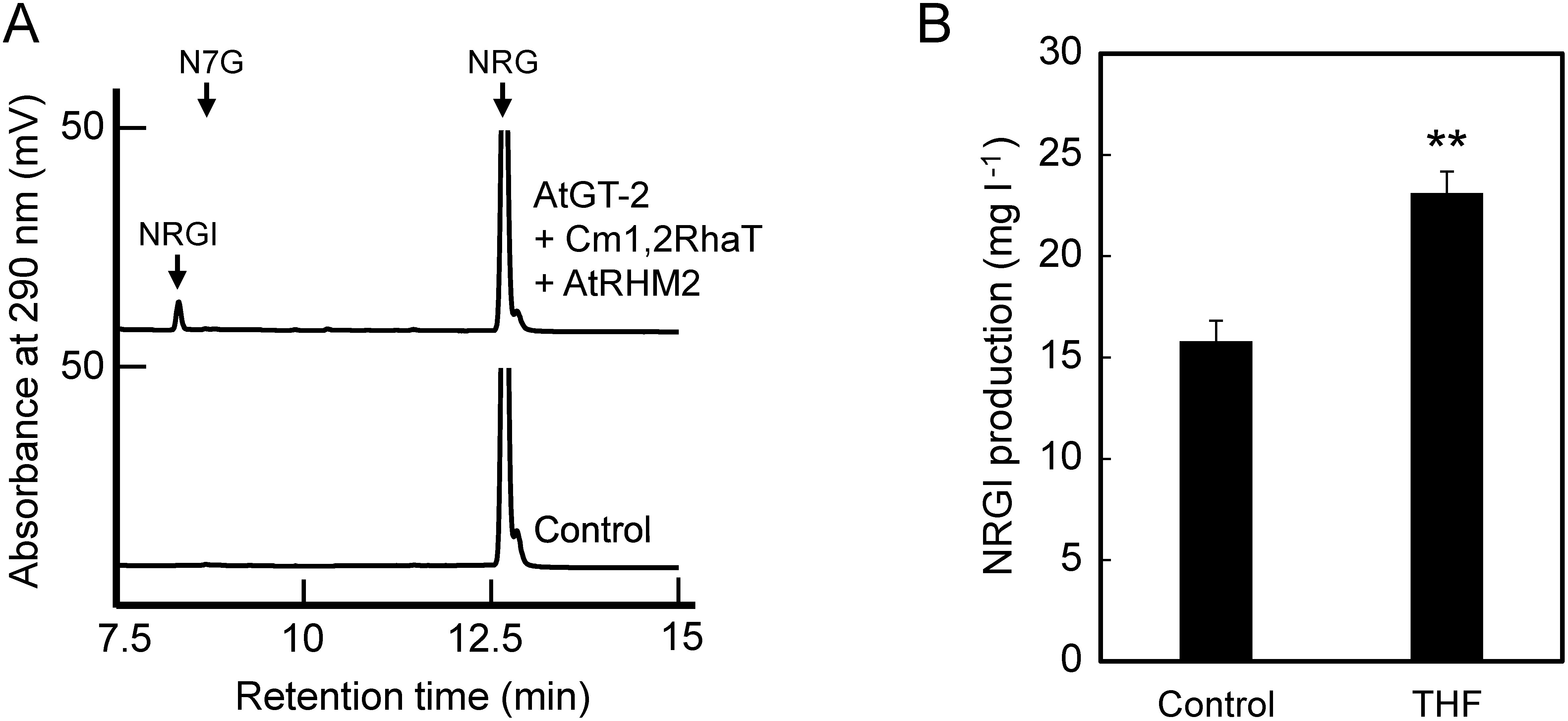
Figure 6. Biotransformation of NRG for the production of NRGI via sequential glycosylation in engineered *S. pombe*. (A) HPLC analysis of the biotransformation products from culture supernatants of *S. pombe* cells harboring either pAUR224-AtGT-2, pREP1-Cm1,2RhaT-MycHis, and pREP2-AtRHM2-MycHis or pAUR224, pREP1-MycHis, and pREP2-MycHis (control). Arrows on the chromatograms indicate the elution positions of NRGI, N7G, and NRG, respectively. All chromatograms are on the same y-axis scale. (B) Effects of THF on NRGI production using *S. pombe* cells harboring pAUR224-AtGT-2, pREP1-Cm1,2RhaT-MycHis, and pREP2-AtRHM2-MycHis. Amounts of NRGI produced in the supernatant after 24 h reactions were measured by HPLC. Control represents biotransformation medium not containing THF. Statistical analysis was performed using an unpaird Welch’s *t*-test, with significant differences indicated by ** *p*<0.01.

We confirmed in vitro NRGI production using the prepared crude soluble protein from the *S. pombe* A8 strain co-expressing AtGT-2, Cm1,6RhaT, and AtRHM2. Next, we attempted in vivo NRGI production from NRG using this strain as a whole-cell biocatalyst in phosphate buffer with an initial cell concentration of OD_600_=33. After 24 h reaction, the supernatant was collected and the biotransformation product was analyzed by HPLC. The results demonstrated that sequential glycosylation of NRG occurred in the co-expressing *S. pombe* A8 transformant but not in the control transformants (Figure 6), and that 15.8±1.0 mg l^−1^ of NRGI was produced in the supernatant. To improve the titer of NRGI by biotransformation via sequential glycosylation, 0.5% (w/w) of THF was supplemented in the biotransformation medium. The highest NRGI titer was achieved at 24 h (23.1±1.1 mg l^−1^, 8.0±0.4% molar conversion) (Figure 6B).

## Discussion

In this study, flavonoid *O*-diglycoside NRGI was successfully produced for the first time via sequential glycosylation using an engineered *S. pombe* strain expressing two UGTs and RHM. To improve the titer, the biotransformation method was developed.

Several studies have reported flavonoid glycoside production in engineered microorganisms. For monoglycosides, optimized conditions yielded 75 mg l^−1^ of N7G in *E. coli* (Dorjjugder and Taguchi 2022) and 87 mg l^−1^ in *S. cerevisiae* (Werner and Morgan 2010). In contrast, diglycoside production generally required strain engineering, such as deletion of genes involved in competing flavonoid glycoside production and optimization of UDP-sugar biosynthetic pathway. For example, in *E. coli*, yields improved to 65 mg l^−1^ of quercetin 3-*O*-α1,2-xylosyl-glucoside and 119.8 mg l^−1^ of rutin (An et al. 2016). In *S. cerevisiae*, 131.3 mg l^−1^ of eriocitrin was obtained (Xiao et al. 2023). In our study, production levels were 31.6 mg l^−1^ for N7G and 23.1 mg l^−1^ for NRGI, lower than those in *E. coli* and *S. cerevisiae*. Nonetheless, we selected *S. pombe* as the host because its eukaryotic cellular environment more closely resembles that of higher organisms, which may be essential for the activity of certain plant-derived enzymes. Adopting strain modification and enzyme engineering could enhance production in future work.

Compared to monoglycosylation, diglycosylation efficiency depends on the availability of the first-step product. NRGI accumulated more when N7G was supplied as substrate (Figure 5C) than when it depended on NRG through the first glycosylation (Figure 5B), indicating that the second glycosylation proceeds in a quantitative manner and the first glycosylation has been identified as the rate-limiting step. Another bottleneck was degradation of products by endogenous β-glucosidases, as indicated by the apparent enhanced enzymatic activity with glucosidase inhibitor (Figure 2D) and the decrease of the titers of N7G in the third batch of the repetitive batch biotransformation reactions (Figure 4D). It is likely that cell lysis and subsequent release of endogenous β-glucosidases were occurred in these later batches, explaining the decline in N7G titers along with the decrease in OD_600_ values. Indeed, in the *S. pombe* genome database (PomBase, Rutherford et al. 2024), 5 β-glucosidase genes are annotated. Among them, *Spexg1*^+^, *Spexg2*^+^, and *Spexg3*^+^ genes were functionally characterized, and their gene products were identified as β-glucosidases (Dueñas-Santero et al. 2010). In addition, the gene deletions of their *S. cerevisiae* homolog, the *ScEXG1* gene, and other *S. cerevisiae* endogenous β-glucosidase genes (*ScSPR1* and *ScEGH1*) resulted in enhanced flavonoid glucoside production (Schmidt et al. 2011; Wang et al. 2016; Xiao et al. 2023). Therefore, the deletions of the *Spexg1*^+^, *Spexg2*^+^, and *Spexg3*^+^ genes may suppress the degradation of the glucosylated flavonoid products and improve their titers.

Furthermore, optimization of culture conditions also affected yields. Phosphate buffer combined with higher cell density enhanced flavonoid glycoside titers compared with YES medium (Figure 4C, Ohashi et al. 2016). However, excessive density (OD_600_=66) caused cell death and competition from increasing endogenous glucosidases. Mechanistically, phosphate buffer may redirect carbon flux toward UDP-Glc generation at the expense of glycolysis, thereby leading to UDP-Glc accumulation that drives flavonoid *O*-glycoside production (de Carvalho 2017). Accordingly, phosphate buffer is a practical alternative to nutrient media when optimal conditions for flavonoid-glycoside production (e.g., pH, temperature, ionic strength) differ from those for cell growth (de Carvalho 2017). Metabolomics-based flux analysis should identify leverage points to increase UDP-Glc supply and, consequently, UDP-Rha biosynthesis, enabling more efficient NRG glycosylation.

Biotransformation products were not accumulated in the cell but were exclusively secreted into the culture supernatant (Figure 4A, B). These observations suggest uptake of NRG, intracellular glycosylation, and secretion of glycosides. No detection of the recombinant AtGT-2-FLAG in the separated supernatant (Supplementary Figure S2) support this proposed process. Permeability reagents enhanced titers (Figure 4E), likely by facilitating substrate uptake, product efflux, and possibly improving NRG solubility, thereby promoting transport across the cell membrane. In addition to passive diffusion, active transport mechanisms may also contribute to the efflux of flavonoids. The additional glucanase treatment and/or the overexpression of transporters responsible for the influx and efflux of the flavonoid aglycones and glycosides may be effective for the improvements of the titers of the glycosylation products. Importantly, incorporating NRG biosynthetic genes (Koopman et al. 2012) into the engineered *S. pombe* provides a framework for sustainable production of flavonoid *O*-diglycosides directly from simple metabolites such as phenylalanine or Glc. Collectively, these strategies including strain engineering, pathway extension, and transport optimization represent powerful avenues to raise yields.

We described here the first example of the biotransformation in engineered *S. pombe* of the flavonoid aglycone to produce the flavonoid *O*-diglycoside via sequential glycosylation. For the biotransformation, a pathway for generating UDP-sugars was introduced into *S. pombe*, which consequently enabled production of diverse flavonoid glycosides. In addition, by altering the glycosyltransferase introduced into this production system, various plant-derived flavonoid glycosides or non-natural flavonoid glycosides can be produced.

## Abbreviations

1,2RhaT: α1,2-rhamnosyltransferase
7-*O*-GlcT: 7-*O*-glucosyltransferase
AbA: Aureobasidin A
AtGT-2: 7-*O*-GlcT from *Arabidopsis thaliana*
AtRHM2: UDP-Rha synthase from *A. thaliana*
Cm1,2RhaT: 1,2RhaT from *Citrus maxima*
Glc: glucose
MM: minimal medium
N7G: naringenin-7-*O*-glucoside
NRG: naringenin
NRGI: naringin
ORF: open reading frame
Rha: rhamnose
rutinoside: α1,6-rhamnosyl-glucoside
RP-HPLC: reversed-phase high-performance liquid chromatography
UGTs: uridine diphosphate sugar-dependent glycosyltransferases

## Description of supplementary files

Supplementary Figure S1. Experiment on the initial cell concentration in phosphate buffer.

Supplementary Figure S2. Immunoblot analysis of recombinant AtGT-2-FLAG protein.
