# Supplementary material for: Production of flavonoid O-diglycoside naringin via sequential glycosylation in engineered Schizosaccharomyces pombe as a whole-cell biocatalyst
Source: Plant Biotechnol (Tokyo). 2026 Jun 25;43(2):181–91. doi: 10.5511/plantbiotechnology.26.0125a (PMC13324211; doi:10.5511/plantbiotechnology.26.0125a)
Supplement: Supplementary Data [file plantbiotechnology-43-2-26.0125a-s001.pdf]

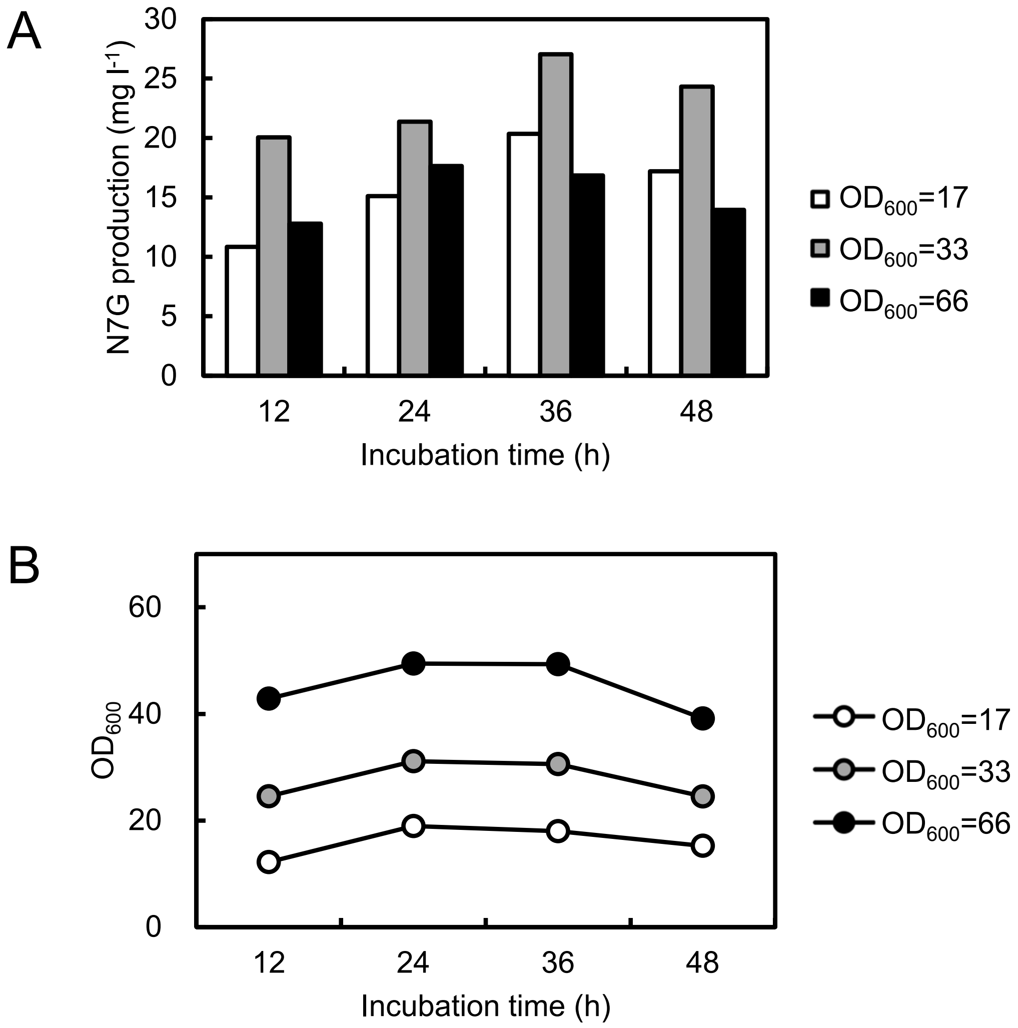

Supplementary Figure S1 Experiment on the initial cell concentration in phosphate buffer (A, B). The initial cell concentration was examined at OD_{600}=17, 33, and 66. The amount of N7G produced in each supernatant was measured by HPLC (A). OD_{600} was valued during the incubation (B). Experiments were conducted with n=2 and the mean values were graphed.

Supplementary Figure S1, Kubomura *et al*.

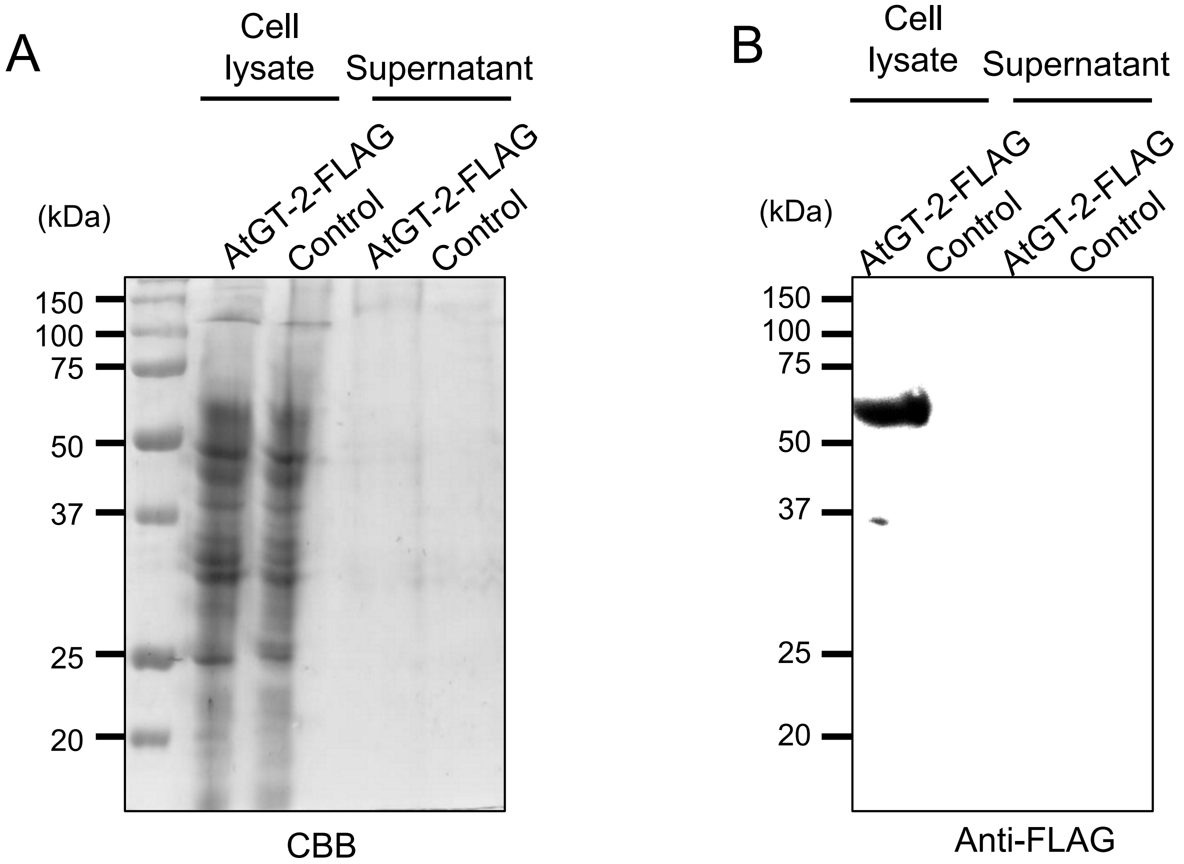

Supplementary Figure S2 Immunoblot analysis of recombinant AtGT-2-FLAG protein. Crude lysate and sulture supernatant from *S. pombe* cells harboring pAUR224-AtGT-2-FLAG or pAUR224 (control) was subjected to 10 % SDS-PAGE and detected by either Coomassie brilliant blue (CBB) staining or immunoblotting using anti-FLAG antibody.

Supplementary Figure S2, Kubomura *et al*.
